# Obesity and Psychopathology From Childhood to Adolescence: A Systematic Review of Prospective Studies

**DOI:** 10.62641/aep.v53i6.1897

**Published:** 2025-12-17

**Authors:** Lucía Beltrán-Garrayo, Blanca Quirós, Ana Rosa Sepúlveda

**Affiliations:** ^1^Department of Biological and Health Psychology, Faculty of Psychology, Autonomous University of Madrid, 28049 Madrid, Spain

**Keywords:** obesity, depression, anxiety, feeding and eating disorders, longitudinal studies, systematic review

## Abstract

**Background:**

Obesity and psychological symptoms often coexist during childhood and adolescence. Understanding the long-term associations between these conditions is essential for designing effective prevention and intervention strategies. The study presented here aimed to examine recent evidence concerning the longitudinal relationship between obesity and commonly comorbid psychopathologies such as depression, anxiety, and eating disorders during childhood and adolescence.

**Methods:**

A systematic review of prospective observational studies was conducted using the PubMed, Scopus, and EBSCOhost databases, covering publications from 2010 to 2025. Twenty-one high-quality studies met the inclusion criteria, especially of participants aged six to 18 years and follow-up periods of at least two years.

**Results:**

A narrative synthesis of 21 prospective studies reveals key longitudinal associations between obesity and psychological symptoms throughout childhood and adolescence, with gender-specific patterns becoming more evident during the teenage years. The collective evidence suggests a bidirectional relationship between eating disorders and obesity. Furthermore, there is evidence indicating a potential prospective association between elevated weight trajectories and depressive symptoms, a connection that appears to be influenced by gender. Specifically, the association from depression to obesity may be more pronounced in older children, particularly girls. Due to the limited number of studies focusing on anxiety symptoms, definitive conclusions regarding their relationship with obesity remain elusive.

**Conclusions:**

This systematic review points to the scarcity of prospective studies that extend beyond two years of follow-up to explore the bidirectional association between obesity and psychological symptoms in youth. While the findings of the present study indicate mutual influences, particularly for eating disorders and depressive symptoms, further research involving longer follow-up periods and diverse populations is warranted. The present study’s results emphasise the importance of early, integrated prevention efforts that address common risk factors.

## Introduction

Obesity is a prevalent health concern in childhood and adolescence [1, 2]. Its multifactorial and complex etiology involves genetic, metabolic, neuroendocrine, environmental, behavioral, and emotional factors [3, 4, 5]. The World Health Organization classifies obesity as a chronic condition that, once established, significantly impacts physical and psychological health [6].

Psychopathological clustering of anxiety, depression, and binge eating is common among individuals with obesity [7], further compounding the burden of excess weight. Studies in youth and adult indicate a bi-directional association between obesity and these psychological disorders [8, 9, 10, 11, 12, 13]. However, this association has been less extensively studied in childhood [14]. Given the high comorbidity of obesity and psychological symptoms from an early age [15, 16], studies suggest that psychopathology (anxiety, depression, impulsivity) may coexist with or precede childhood obesity, contributing to an obesogenic environment [17, 18]. Children with overweight and obesity also show a higher prevalence of psychological diagnoses than their normal-weight peers (48% vs. 2%) [19]. This raises the question of whether a reciprocal link between obesity and psychiatric disorders exists from childhood to adolescence.

The association between depressive symptoms and obesity in childhood is well established, but its directionality remains unclear [20]. Blaine [21], in a meta-analysis of longitudinal studies including adolescents and adults, found that depression increased the risk of later obesity. Incledon *et al*. [22] and Liem *et al*. [23], in systematic reviews of children and adolescents, reported similar evidence for depression predicting subsequent overweight or obesity. Mannan *et al*. [24], in a systematic review and meta-analysis, suggested that bidirectional associations may emerge in adolescence, whereas Mühlig *et al*. [25], in a systematic review focused on childhood and adolescence, found inconsistent results, with only a minority of studies supporting effects in either direction. Overall, depression appears to be a consistent risk factor for later obesity, while evidence for bidirectionality is limited to adolescence and remains inconclusive in childhood.

Fewer studies have examined the comorbidity between obesity and anxiety [9]. However, a meta-analysis by Burke & Storch [26] concluded that this association is significant during childhood and adolescence, though, the limited number of longitudinal studies prevents determining the directionality.

Common eating disorders in adolescents with obesity include binge eating disorder, bulimia nervosa, and, to a lesser extent, atypical anorexia nervosa [27]. A recent narrative review suggests a possible bidirectional relationship between obesity and eating disorders beginning in childhood [28], but to our knowledge, this relationship has not yet been systematically reviewed.

In summary, existing literature suggests a plausible bidirectional association between obesity and psychological disorders from an early age. However, no studies have systematically reviewed the prospective relationship between multiple psychological disorders and obesity in this age group. Identifying whether these conditions serve as mutual risk factors may inform prevention and early intervention strategies. Thus, this study aims to synthesize high-quality research on the longitudinal relationship between obesity and psychological symptomatology in childhood and adolescence. Specifically, we address the following questions:

Does the presence of depressive, anxiety, and eating disorder symptoms in childhood increase the risk of obesity in adolescence?

Does childhood obesity increase the risk factor of developing depressive, anxiety, and eating disorder symptoms in adolescence?

## Methods

This study followed the Preferred Reporting Items for Systematic Reviews and Meta-Analyses (PRISMA) guidelines [29]. The research question, inclusion criteria, and search terms were defined using the Population, Intervention, Comparison, Outcome (PICO) approach [30]. The review protocol was registered in PROSPERO (CRD42021249042).

### Search Procedure and Strategy

A comprehensive literature search was conducted using the PubMed, Scopus, and EBSCOhost databases in two phases. Because previous studies addressing these questions lacked conclusive findings at this developmental stage [11, 26], the search was restricted to publications from 2010 onward.

The first search phase, conducted in 2022, covered publications from 2010 to May 2022. The search strategy (see Table 1) combined terms related to high weight status (e.g., obesity, overweight, high body mass index), psychological symptomatology (e.g., mental disorder, psychiatric diagnosis, psychological symptoms), and prospective study designs (e.g., longitudinal, long-term, follow-up). Search strategies were designed to exclude intervention studies and retain only prospective observational research.

**Table 1.** **Databases, search equation and number of records per database**.

Databases	Search Equation	Records
PubMed	(obes*[Title] OR overweight[Title] OR Body Mass Index[Title] OR BMI[Title]) AND (longitudinal*[Title] OR prospective*[Title] OR follow-up[Title] OR trajector*[Title]) AND (Mental Disorders[MeSH Terms] OR mental disorder[Text Word] OR depression[MeSH Terms] OR depress*[Text Word] OR anxi*[Text Word] OR anxiety[MeSH Terms] OR Binge-Eating Disorder[MeSH Terms] OR Eating disorder*[Text Word] OR psychiatric disorder*[Text Word] OR psychological disorder[Text Word]) Filters: Free full text, Full text, English, Spanish, from 2010–2022.	First search: 163 Second search: 50
Scopus	((TITLE-ABS-KEY (“mental disorder”) OR TITLE-ABS-KEY (depression) OR TITLE-ABS-KEY (anxiety) OR TITLE-ABS-KEY (“binge eating disorder”) OR TITLE-ABS-KEY (“eating disorder”) OR TITLE-ABS-KEY (“psychiatric disorder”) OR TITLE-ABS-KEY (“psychological disorder”)) AND PUBYEAR > 2009) AND ((TITLE-ABS-KEY (adolescen*) OR TITLE-ABS-KEY (child*)) AND PUBYEAR > 2009) AND ((TITLE (longitudinal) OR TITLE (prospective) OR TITLE (traject*) OR TITLE (follow-up)) AND PUBYEAR > 2009) AND ((TITLE (obes*) OR TITLE (“Body mass index”) OR TITLE (bmi)) AND PUBYEAR > 2009.)	First search: 107 Second search: 40
EBSCOhost	TI (obesity or overweight or fat or obese or unhealthy weight or high bmi or body mass index or BMI) AND TI (prospective* or longitudinal or traject*) AND AB (depression or depress* or depressive disorder or depressive symptoms or major depressive disorder or anxiety or anxious or anxi* or eating disorder or binge eating or mental disorder or psychiatric disorder or psychological disorder) NOT (treatment or intervention or therapy or management or rehabilitation) NOT (pregnancy or pregnant or prenatal or antenatal or perinatal or maternal). Filters: from 2010–2022.	First search: 161 Second search: 62

Notes: MeSH Terms, Medical Subject Headings Terms; TITLE-ABS-KEY, Scopus search fields “Title, Abstract, and Keywords”; PUBYEAR, Scopus publication year filter; TI, EBSCOhost “Title” search field.

To update the findings, a second search phase was performed in February 2025, following the same methodology and inclusion criteria. This search covered studies published between May 2022 to February 2025. Additionally, a manual reference check of the included articles identified one additional relevant study.

### Inclusion and Exclusion Criteria

The inclusion criteria were as follows:

(a) Were published in English or Spanish.

(b) Were published between 2010 and 2025.

(c) Were prospective observational studies.

(d) Analyzed the prospective association between obesity and psychological symptomatology, or vice versa

(e) Had a minimum follow-up of two years.

(f) Included participants aged 6 to 18 in at least two follow-up assessments.

(g) Had a sample size of at least 100 participants.

(h) Used standardized cut-off points for Body Mass Index (BMI).

(i) Employed validated psychological assessment instruments.

We excluded: (a) studies involving participants receiving weight loss treatment, (b) studies including participants with chronic medical conditions (e.g., autoimmune or metabolic diseases) or using medication that could affect weight or psychological health.

### Categorization of Variables

Obesity screening is primarily based on BMI [2]. In children and adolescents aged 5–19 years, obesity is defined as a BMI greater than 2 standard deviations above the WHO Growth Reference median for sex and age [6]. This review includes both quantitative weight status measures (e.g., sex- and age-standardized BMI (z-BMI), changes in BMI or z-BMI) and qualitative measures (e.g., categories based on z-BMI) as well as transitions between weight categories (e.g., from overweight to obesity).

Psychological symptomatology in this review refers to the risk or presence of depressive, anxiety, and eating disorders. Psychological outcomes were reported heterogeneously across studies. To summarize findings consistently, we grouped them into four, non–mutually exclusive categories: (1) eating disorder symptomatology; (2) depressive symptoms; (3) anxiety symptoms; (4) internalizing symptoms, where studies assessed anxiety and depression jointly using combined scales. This categorization reflects the way constructs were operationalized in the original studies rather than a predefined taxonomy.

### Study Selection

Two independent reviewers (L.B. and B.Q.) manually screened titles and abstracts, selecting eligible studies through consensus. Next, the same reviewers assessed the full texts of the selected articles. Any disagreements regarding eligibility were resolved through discussion or with the assistance of a third reviewer (A.R.S.).

### Data Extraction and Synthesis

A data extraction table was created to capture key study elements including: authors, year, country, sample size (% male gender), age at baseline, duration of follow-up in years, number of evaluations, percentage of sample loss from baseline, main research questions concerning the systematic review, predictor variable, response variable, covariates, and results. One reviewer (B.Q.) entered the data into the extraction table, and a second reviewer (L.B.) verified its accuracy. Given the heterogeneity of statistical approaches and outcome measures across studies, extracted data were analyzed based on their relevance to each study’s methodology. The results are presented as a narrative synthesis to provide a coherent overview of the evidence. A meta-analysis was not performed due to the heterogeneity of study designs.

### Quality Assessment

Potential sources of bias were evaluated using an adapted version of the Newcastle-Ottawa Scale (NOS) for prospective observational cohort studies [31] (see Appendix Table 5). This scale assesses sample selection, comparability, and outcome, with a maximum score of 8 per study. Two independent researchers (L.B. and A.R.S) conducted the quality assessment, resolving discrepancies through discussion.

## Results

### Inclusion of Studies

**Fig. 1.**
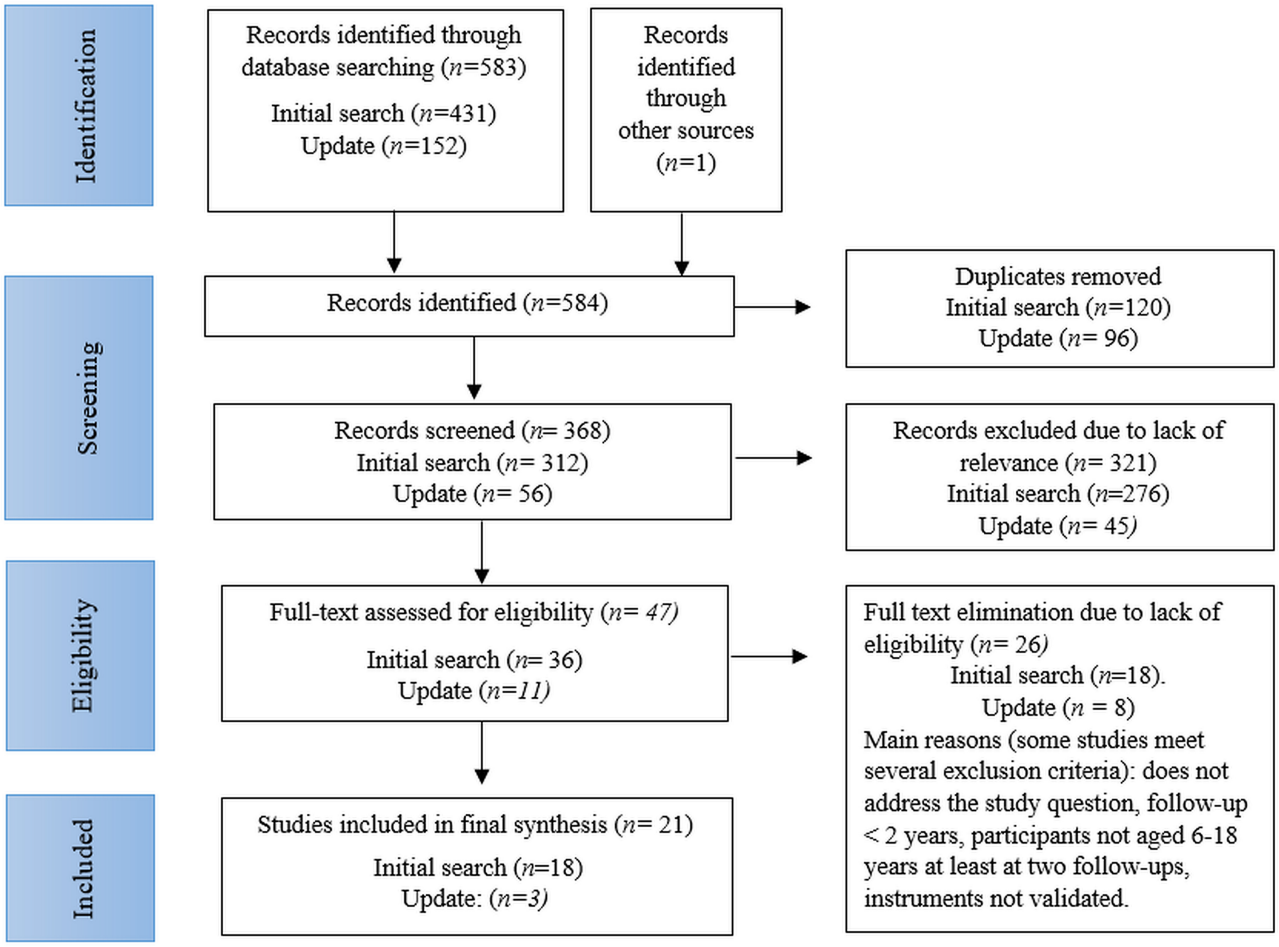
**Preferred Reporting Items for Systematic Reviews and Meta-analyses (PRISMA) flow diagram of the literature search and screening process**.

Fig. 1 presents the study selection process. After removing duplicates, the initial search yielded 312 records, with one additional study identified through reference searching. The updated search (covering studies published since 2022) retrieved an additional 152 records.

Following title and abstract screening, 321 records were excluded due to irrelevance (276 from the initial search, 45 from the update). Among the full-text articles assessed, 18 from the initial search and 8 from the update were excluded, primarily due to not addressing the research question, the follow-up duration of less than 2 years, and participants not aged 6–18 years at least at two follow-ups. Ultimately, 21 studies were included in the qualitative synthesis.

### General Characteristics of the Studies

Table 2 (Ref. [32, 33, 34, 35, 36, 37, 38, 39, 40, 41, 42]) presents the characteristics of studies assessing psychological symptomatology as a risk factor for weight gain or obesity (n = 11/21). Table 3 (Ref. [32, 39, 40, 43, 44, 45, 46, 47, 48, 49, 50, 51, 52]) includes studies analyzing obesity or weight gain trajectories as a risk factor for psychological symptomatology (n = 13/21). Three studies [32, 39, 40] assess the relationship bidirectionally and were included in both tables.

**Table 2.** **Psychological symptoms to BMI: characteristics and main findings of the included studies**.

Authors (year), Country	Sample details	Follow-up details	Psychological symptoms measurement	Outcome: weight status (measurement)	Cofounder variables	Main findings
Ames & Wintre (2016) [32], Canada	N = 6987 (50.6% males), Baseline: 10–11 y.	Duration: 8 y. 4 assessments. Dropout not described.	Internalizing symptoms (anxiety and distress): Behaviours NLSCY Checklist (Statistics Canada, 1995).	BMI and change in BMI. (SR/SR-P)	Gender, SES	For girls, internalizing symptoms at 10 were positively associated with a more rapid increase in BMI (b = 0.02, SE = 0.01, *p* = 0.02) and higher BMI in adolescence (b = 0.230, SE = 0.057, *p * < 0.001). For boys, internalizing symptoms did not predict BMI or change in BMI (*p * > 0.05).
Anderson *et al*. (2011) [33], United States	N = 918 (0% males), Baseline: 12 y.	Duration: 2 y. 2 assessments. Dropout not described.	Depressive symptoms CES-D (Radloff, 1991).	Obesity, based on BMI. (M)	Age, Baseline obesity, Ethnicity, SES, Time spent alone at home	Depressive symptoms at 12 years were associated with a greater likelihood of obesity at 14 years. This association was higher for white girls (*p * < 0.02). After adjusting for baseline obesity, the interaction with ethnicity was no longer significant. The odds ratio for white girls remained significant (OR = 3.68, CI: 1.72, 7.87), but not for Hispanic and black girls.
Bjornelv *et al*. (2011) [34], Norway	N = 1619 (46.1% males), Baseline: 13–16 y.	Duration: 3 y. 2 assessments. 20% drop-out.	Eating problems: EAT-7 (Bjomelv, 2002). Emotional symptom (anxiety and depression) SCL-5 (Strand *et al*., 2003; Tambs y Moum, 1993).	Z-BMI trajectories: (1) Healthy change: from weight problems (underweight, overweight, obesity) toward normal weight, (2) Unhealthy change: from normal weight to weight problems. (M)	Age, Gender, Inactivity, Personality Traits, Self-esteem, Sex, Smoking	Eating symptoms: low degrees of self-control about eating at baseline predicted an unhealthy weight increase in both sexes (OR = 0.6, CI: 0.4–0.9), but also a healthy weight increase in boys (OR = 0.2, CI: 0.04–0.9).
						Emotional symptoms did not predict weight change during adolescence (*p * > 0.05).
Cho *et al*. (2018) [35], United States	N = 3262 (46.7% male), Baseline: 14–15 y.	Duration: 2 y. 5 assessments. 4.8% drop-out.	Depressive symptoms: CESD (Radloff, 1991).	BMI trajectories: stable normative weight; overweight to normative weight, overweight to chronically obese, normative weight to overweight. (SR)	Age, Anhedonia, Ethnicity, Gender, Parental education	Baseline depression was not associated with any BMI trajectory group (*p * > 0.08).
Christiansen *et al*. (2017) [36], United States	N = 422 (49.3% male), Baseline: 8 y.	Duration: 3 y (11 years for parents covariables). 2 assessments (11 assessments for parental covariates). 40.4% drop-out.	Eating Behaviour: EPI-C (Schacht *et al*., 2006). Depressive symptoms: DIKJ (Stiensmeier-Pelster *et al*., 2014).	Z-BMI categories: under-/normal weight, overweight and obesity. (M)	Baseline Z-BMI, Gender, General psychopathology, IQ, PA, Parents BMI, SES	Restrained eating via BMI at eight years and parental BMI showed a weak association with BMI at age 11 for males and females. External and emotional eating did not show a prospective association.
						Depressive symptoms did not predict later BMI (*p * > 0.05).
Kubzansky *et al*. (2012) [37], United States	N = 1528 (48.8% male), Baseline: 12 y.	Duration: 4 y. 4 assessments. 21.3% drop-out.	Depressive symptoms: CESD (Radloff, 1991). Anxiety symptoms: STAI (Spielberger *et al*., 1983).	BMI trajectories: (1) persistent normal weight, (2) persistent overweight, (3) from obesity to overweight, (4) persistent obesity, and (5) persistent severe obesity. (M)	Age, Ethnicity, Gender, SES	Depressive symptoms were associated with higher BMI trajectories. Example of wave 3: Obesity (OR: 1.27, CI = 1.06–1.51), severe obesity (OR: 1.43, CI = 0.95–2.17).
						Anxiety symptoms were associated with higher BMI trajectories. Example of wave 3: Obesity (OR: 1.33, CI = 1.08–1.62), severe obesity (OR: 1.17, CI = 0.71–1.92).
						Note that normal weight was the reference BMI trajectory.
Larsen *et al*. (2014) [38], Netherlands	N = 2051 (51.5% male), Baseline: 12 y.	Duration: 3 y. 3 assessments. 28.58% drop-out.	Depressive symptoms: CESD (Radloff, 1991).	Z-BMI. (M)	Baseline Z-BMI, Education, Gender, Menarche, Smoking	Depressive symptoms were not associated with an increase of z-BMI (slope), neither in the total sample nor for boys or girls (*p * > 0.05).
Marmorstein *et al*. (2014) [39], United States	N = 1512 (49.7% male), Baseline: 11 y.	Duration: 13 y. 5 assessments. 10% average drop-out per wave.	Major Depresive Disorder: Diagnostic Interview Schedule for Children and Adolescents (Reich and Welner, 1988).	Obesity onset, based on BMI. (M)	Age, Gender	A major depressive disorder that developed by age 14 was associated with the onset of obesity in late adolescence among females but not males. Males (OR: 0.33, CI = 0.05–2.36), females (OR: 3.76, CI = 1.33–10.59).
Patalay and Hardman (2019) [40], United Kingdom	N = 15,369 (51.2% male), Baseline: 3 y.	Duration: 11 y. 5 assessments. Drop-out 25.5%.	Internalizing symptoms: SDQ (Goodman, 1997).	BMI. (M)	Age, Baseline BMI, Ethnicity, Gender, SES	Co-development of BMI and internalizing symptomatology from 7–14 years (r = 0.23; *p * < 0.001). No gender differences were found. Controlling for SES, internalizing symptoms did not predict BMI from 7 to 11 years, but it did from 11 to 14.
Rehkopf *et al*. (2011) [41], United States	N = 2379 (0% male), Baseline: 9–10 y.	Duration: 10 y. 10 assessments 10% drop-out.	Eating problems: EDI-C (Garner *et al*., 1983). Anxiety symptoms (Reynolds y Richmond, 1978).	BMI percentile changes, Overweight/obesity onset (based on BMI). (M)	39 measures within these categories: dietary intake, eating behaviors, PA, psychological, social, and parental health	Eating problems: drive to restrict predicted change in BMI percentile (*p * < 0.05) but was not associated with the onset of overweight or obesity. Emotional eating predicted change in BMI percentile and obesity onset (*p * < 0.05) but did not predict overweight onset.
						Anxious symptoms predicted a change in BMI percentile (*p * < 0.05) but were not associated with the onset of overweight or obesity.
Wade *et al*. (2017) [42], Belarus	N = 13,557 (51.8% male), Baseline: 6.5 y.	Duration: 16 y. 3 assessments. 20.5% drop-out.	Eating problems: ChEAT (Garner y Garfinkle, 1979).	z-BMI, Overweight/obesity onset (based on z-BMI) (M)	Adiposity at baseline, Age, Baseline Z-BMI, Gender, SES	After controlling for baseline SES, problematic eating attitudes at 11.5 years predicted obesity onset (OR: 2.18; CI = 1.58, 3.02). This association remained significant after controlling for baseline z-BMI (OR: 1.80; CI = 1.28, 2.53).

Notes: AN, Anorexia Nervosa; BE, Binge Eating; BMI, Body Mass Index; BN, Bulimia Nervosa; CESD, Center for Epidemiologic Studies Depression Scale; CHEAT, Children’s Eating Attitudes Test; DIKJ, Depression Inventory for Children; EAT, Eating attitudes test; EDI-C, Eating Disorder Inventory Children; EPI-C, Eating Pattern Inventory for Children; IQ, intelligence quotient; M, measured by a professional; OR, Odds Ratio; PA, Physical activity; SCL-5, Symptom Checklist-5; SDQ, Strengths and Difficulties Questionnaire; SE, Standard Error; SES, socioeconomic status; SR, Self-reported; SR-P, Self-reported parents; STAI, State-Trait Anxiety Inventory.

**Table 3.** **BMI to psychological symptoms: characteristics and main findings of the included studies**.

Authors (year), Country	Sample details	Follow-up details	Weight status variable/measurement	Psychological variables: instruments	Cofounder variables	Main findings
Al-Shoaibi *et al*. (2024) [43], United States	N = 9964 (51.4% male), Baseline: 9–13 y.	Duration: 3 y. 4 assessments. % Drop-out not reported.	BMI percentile. (M)	Binge eating symptoms: KSADS-5 (parent-reported, DSM-5 criteria).	Age, Gender, Ethnicity, SES	Higher BMI at baseline was significantly associated with increased risk of developing BE in both adolescents with (HR = 1.03, 95% CI 1.00–1.06) and without (HR = 1.05, 95% CI 1.03–1.07) binge-eating behavior.
						Adolescents with BMI ≥85th percentile had a significantly higher risk of BE onset compared to those with BMI <85th percentile: HR = 2.60 (95% CI 1.00–6.68) for those with binge-eating behavior at baseline. HR = 6.01 (95% CI 3.90–11.10) for those without binge-eating behavior at baseline.
Ames and Wintre (2016) [32], Canada	N = 6987 (50.6% male), Baseline: 10–11 y.	Duration: 8 y. 4 assessments. % Drop-out not reported	BMI. (SR-P/SR)	Internalizing symptoms (anxiety and distress): Behaviours NLSCY Checklist (Statistics Canada, 1995). Depressive symptoms: CES-D (Radloff, 1977).	Gender, SES	Girls: BMI at age 10 was associated with a faster internalizing symptom increase (b = 0.074, SE = 0.019, *p * < 0.001), and with higher internalizing symptoms (b = 0.012, SE = 0.004, *p * < 0.003) at ages 16 and 17 years. BMI at age 10 was associated with higher levels of depressive symptoms at ages 16 and 17 (b = 1.515, SE = 0.476, *p* = 0.001).
						Boys: BMI at age 10 was associated with a faster internalizing symptom increase (b = 0.045, SE = 0.012, *p * < 0.001), and with higher internalizing symptoms (b = 0.009, SE = 0.003, *p * < 0.001) at ages 16 and 17 years. No association was found with depressive symptoms by the CES-D.
Beltrán-Garrayo *et al*. (2023) [44], Spain	N = 100 (50 obesity, 51 normal weight) (46.5% male), Baseline: 8–12 y.	Duration: 5 y. 2 assessments. 29% drop-out.	BMI. (M)	Depressive symptoms: CDI (Kovacs, 1992). Anxiety symptoms: STAIC (Spielberger *et al*., 1973). Eating problems: ChEAT (Garner y Garfinkle, 1979).	Baseline BMI, Baseline psychological symptoms, Gender, Parental psychopathology, SES	Higher BMI z-score at baseline was associated with greater eating symptomatology at follow-up (β = 0.28, *p * < 0.05).
						Eating symptoms at baseline were a significant predictor of later clinical diagnosis in the obesity group (β = 0.11, *p* < 0.05).
						No significant longitudinal associations were found between baseline BMI and later depressive or anxiety symptoms.
Blundell *et al*. (2024) [45], United Kingdom	N = 13,135 (50.4% male), Baseline: 7 y.	Duration: 7 y. 3 assessments. 29.2% drop-out.	BMI. (M)	Depressive symptoms: Short Mood and Feelings Questionnaire (Angold *et al*., 1995).	Baseline BMI, Baseline depressive symptoms, Body dissatisfaction, Gender, SES	Higher BMI at age 7 was associated with greater body dissatisfaction at age 11 (β = 0.21, SE = 0.05, *p * < 0.001).
						Body dissatisfaction at age 11 was a significant predictor of depressive symptoms at age 14 (β = 0.34, SE = 0.07, *p* < 0.001).
						The indirect effect of BMI at age 7 on depressive symptoms at age 14, mediated by body dissatisfaction, was significant (β = 0.07, SE = 0.02, *p* = 0.002).
						After adjusting for mediators, the direct association between BMI at age 7 and depressive symptoms at age 14 was not significant (β = 0.04, SE = 0.03, *p* = 0.08).
Francis *et al*. (2020) [46], United States	N = 1077 (50.3% male), Baseline: 15 months.	Duration: 14 y. 11 assessments. 20.4% drop-out.	BMI trajectory membership: nonoverweight *p * < 40, nonoverweigh *p * < 70, overweight/obesity, severe obesity. (M)	Eating problems: EAT-26 general score and dieting subscale (Garner *et al*., 1982)	Age, Ethnicity, Gender, Inhibitory control at 5 y, Pubertal status, Self-regulation at 5 y	Youth on the severe obesity trajectory presented higher levels of general eating symptomatology at 15 years, followed by youth on the overweight/obese trajectories (*p * < 0.5). Overweight/obesity (OR: 6.9, CI = 5.5–8.2), severe obesity (OR: 7.9, CI = 6.1–9.7). The restraint scale followed the same pattern: overweight/obesity (OR: 2.4, CI = 1.9–3.0), severe obesity (OR: 3.5, CI = 2.6–4.4). Female gender and higher pubertal status were associated with eating dysregulation (*p * < 0.01).
Goodman and Must (2011) [47], United States	N = 102 (51 obesity, 51 normal weight) (33% male), Baseline: 12–17 y.	Duration: 3 y. 3 assessments. 28% drop-out.	Severe Obesity vs normal weigh (based on Z-BMI). (M)	Depressive symptoms: CES-D (Radloff, 1977).	Age, Ethnicity, Gender	Depressive symptoms were higher among adolescents with severe obesity at baseline, the difference being statistically significant at 3-year follow-up (*p* = 0.02). Ethnicity moderated this association, remaining only among non-Hispanics white (*p * < 0.05). No differences were found in the prevalence of severe depressive symptoms using cut-off points at any of the three-time follow-ups (*p * > 0.05).
Hoare *et al*. (2016) [48], Australia	N = 634 (46.7% male), Baseline: 11–14 y.	Duration: 2 y. 2 assessments. 25.5% drop-out.	Weight categories (based on Z-BMI). (M)	Depressive symptoms: Short Mood and Feelings Questionnaire (Angold *et al*., 1995).	Age, Ethnicity, Gender, Parents’ educational level, School attended	Boys: stable overweight or obesity was associated with later depressive symptomatology (b = 1.63, CI = 0.33–2.92).
						Girls: weight status was not significantly associated with subsequent depressive symptomatology (*p* = 0.05).
Huang *et al*. (2013) [49], United States	N: 5156 (56.8% male), Baseline: 6 y.	Duration: 12 y. 7 assessments. 55.15% drop-out.	Obesity trajectory membership (based on Z-BMI percentile): Chronically with Obesity, increasing, decreasing, without obesity. (SR-P/SR)	Depressive symptoms: CES-D (Radloff, 1977)	Age, Delinquency, Ethnicity, Gender, Peer pressure, School experiences, Self-control, Self-esteem, Sexual behaviors, Substance use	The group with chronical obesity and increasing weight groups showed higher levels of depressive symptoms but none were associated with a distinctive obesity trajectory (*p * > 0.05).
Martin-Storey and Crosnoe (2015) [50], United States	N = 957 (49.9% male), Baseline: 1–3 y.	Duration: 13 y. 10 assessments 29.8% drop-out	Overweight/obesity trajectory membership (based on BMI percentile): early childhood onset, middle childhood onset, stably overweight, early childhood limited overweight class, never overweight. (M)	Depressive symptoms: CDI (Kovacs, 1992)	Baseline depressive symptoms, Body Image, Ethnicity, Family structure, Gender, Maternal age, Maternal depression, Self-perceived Popularity Victimization, SESS	Girls within the stably overweight trajectory were the more likely to be depressed at age 15, even after controlling for all cofounders (*p * < 0.01). Boys: weight trajectories were not associated with depressive symptomatology (*p * > 0.05).
Marmorstein *et al*. (2014) [39], United States	N = 1512 (49.7% male), Baseline: 11 y.	Duration: 13 y. 5 assessments.	BMI trajectory. (M)	Major depressive disorder: DSM-III-R, through Diagnostic Interview Schedule for Children and Adolescents (Spitzer *et al*., 1987)	Age, Gender	Obesity onset by 14 years old did not significantly predict the onset of MDD during late adolescence. Obesity that developed in late adolescence (14–20 years old) predicted MDD onset in early adulthood among females (OR = 5.89, CI = 2.31–15.01).
Patalay and Hardman (2019) [40], United Kingdom	N = 15,369 (51.2% male), Baseline: 3 y.	Duration: 11 y. 5 assessments. 25.5% drop-out.	BMI. (M)	Internalizing symptoms: SDQ (Goodman, 1997)	Age, Ethnicity, Gender, SES	Co-development of BMI and internalizing symptomatology from 7–14 years (r = 0.23; *p * < 0.001). No gender differences were found. Controlling for SES, BMI at seven years predicted internalizing symptoms at 11 years (*p * < 0.05), but BMI at 11 did not predict internalizing symptoms at 14 years old.
Pryor *et al*. (2016) [51], Canda	N = 1221 (46% male). Baseline at 6 years for exposure, outcome, and covariates.		Overweigh/obesity trajectory (based on BMI): early-onset, late-onset, never overweight (72.5%). (M)	Depressive symptoms: CDI (Kovacs, 1985). Anxiety symptoms: Child Behavior Questionnaire (Tremblay *et al*., 1991).	Body dissatisfaction, Child mental and physical health, Family adversity, Gender, Peer victimization	Children on an early and late onset overweight/obesity trajectory were at increased risk for depression at 13 years. Early onset to depression (b = 0.318, CI = 0.141–0.496), late onset to depression (b = 0.332, CI = 0.187–0.477), early onset to anxiety (b = 0.262, CI = 0.09–0.44), late onset to anxiety (b = 215, CI = 0.072–0.358).
						Body dissatisfaction mediated the association in both early and late onset; peer victimization was a mediator only for early onset.
Yilmaz *et al*. (2019) [52], United States	N = 1502 (42.2% male), Baseline: birth.	Duration: 18 y. 3 assessments. % drop-out not reported.	BMI trajectories. (SR)	Eating Disorder Diagnosis: DSM-5 criteria	Birthweight Ethnicity, Gender, Maternal age at birth, Maternal history of psychiatric disorders (including ED), Social class	Girls: those who developed restrictive AN had lower BMI trajectory by 4 years (b = −0.505, SE = 0.22), those with BN showed higher BMI trajectories after 2 years (b = 0.74, SE = 0.33), for BED higher BMIs at 6 years (b = 0.88, SE = 0.29), and those who developed purging AN had higher mean BMIs by 5 years (b = 0.58, SE = 0.26). Note that non-ED was the control group.
						Boys: those who developed restrictive AN had lower BMIs by two years (B = −0.87, SE = 0.38), boys who with BED diverged from the no-ED control group at four years (B = 1.03, SE = 0.51), and purging AN showed higher BMIs by six years (B = 1.48, SE = 0.64). No association was found for BN.

Notes: AN, Anorexia Nervosa; BE, Binge Eating; BMI, Body Mass Index; BN, Bulimia Nervosa; CDI, Children’s Depression Inventory; CESD, Centers for Epidemiological Study-Depression; DSM, Diagnostic and Statistical Manual of Mental Disorders; EAT, Eating Attitudes Test; HR, Hazard Ratio; K-SADS-5, Kiddie Schedule for Affective Disorders and Schizophrenia, 5th Edition; SES, Socioeconomic Status; M, measured by a professional; OR, Odds Ratio; SDQ, Strengths and Difficulties Questionnaire; SE, Standard Error; SR, Self-reported; SR-P, Self-reported parents.

All but one study [33] included both male and female participants. The majority of the studies were conducted in the United States (n = 12/21) [33, 35, 36, 37, 39, 41, 43, 46, 47, 49, 50, 52], with two in Canada [32, 51], one in Australia [48], and the remaining in Europe: Belarus [42], Norway [34], the Netherlands [38], Spain [44], and the United Kingdom [40, 45].

Baseline ages ranged from birth to 16 years, with follow-up durations spanning 2 to 18 years. The number of follow-up assessments varied from two to eleven. Key covariates included gender (n = 19/21), age (n = 12/21), socioeconomic status (n = 10/21), and ethnicity (n = 10/21).

### Psychological Symptomatology as a Risk Factor for Weight Gain/Obesity

Four studies examined the influence of eating symptomatology on weight status. Christiansen *et al*. [36] found that restrained eating at age 8, when considering baseline BMI and parental BMI, was associated with BMI at age 11. However, external and emotional eating did not predict BMI.

Bjornelv *et al*. [34] reported that low self-control over eating predicted unhealthy weight gain but also served as a protective factor against unhealthy weight loss. Rehkopf *et al*. [41] assessed 41 potential predictors of weight change and overweight and obesity onset, finding that food restriction predicted changes in BMI percentile but not onset overweight or obesity. Meanwhile, emotional eating was among the main predictors of BMI change and obesity onset from age 9 to 19 [41]. Finally, Wade *et al*. [42] reported that problematic eating attitudes in mid-childhood were associated with later obesity development.

Six studies included specific measures of depressive symptoms, with four reporting prospective associations. Anderson *et al*. [33] conducted a two-year prospective study among 12-year-old girls. Non-adjusted analyses showed that depressive symptoms predicted obesity at follow-up across ethnicities, but after controlling for baseline obesity, this remained true only among white girls. Larsen *et al*. [38] found that among girls—but not boys—depressive symptoms at age 12 showed a weak positive association with z-BMI at 15 years old. However, depressive symptoms did not predict changes in BMI over time for either gender. Marmorstein *et al*. [39] found that the onset of Major Depressive Disorder in early adolescence predicted the onset of obesity in late adolescence for females but not males. Lastly, Kubzansky *et al*. [37] reported that depressive symptoms at 12 years were associated with higher BMI trajectories four years later.

In the remaining two studies, no evidence was found. Christiansen *et al*. [36] found no link between depressive symptoms and weight status in either gender from ages 8 to 11. Similarly, Cho *et al*. [35] found no association between baseline depression at 14–15 and BMI trajectory after two years.

Two studies used a specific anxiety measure. Rehkopf *et al*. [41] found that while anxious symptomatology predicted changes in BMI percentile from age 9 to 19, it was not related to the onset of overweight or obesity. In the study conducted by Kubzansky *et al*. [37], anxiety symptoms prospectively correlated with obesity and severe obesity trajectories.

Three studies assessed anxiety and depressive symptoms jointly using combined scales of internalizing symptoms. Bjornelv *et al*. [34] found no evidence that internalizing symptoms at ages 13–16 predicted changes in weight status after three years. Meanwhile, Ames & Wintre [32] found that for girls—but not boys—internalizing symptomatology at age 10 predicted a faster increase in BMI from that age to 18 years, as well as higher BMI at follow-up. Finally, Patalay and Hardman [40] reported that the longitudinal association between internalizing symptoms and BMI was not significant from ages 7 to 11, but became significant from ages 11 to 14 in both genders.

### Obesity as a Risk Factor for Psychological Symptomatology

Three studies examined the link between obesity and later eating disorder symptoms. Francis *et al*. [46] found that female gender and pubertal status were positively associated with eating dysregulation. After controlling for these confounders, they concluded that adolescents with obesity and overweight trajectories from 15 months to 15 years had higher disturbed eating scores and restrictive eating at age 15. Similarly, Yilmaz *et al*. [52] found that higher BMI trajectories were associated with the development of purging anorexia nervosa, bulimia nervosa, and binge eating disorder. Beltrán-Garrayo *et al*. [44] further supported these findings, reporting that childhood obesity was associated with an increased risk of developing disordered eating behaviors in adolescence, particularly restrictive eating, binge eating, and compensatory behaviors.

Nine studies assessed obesity as a predictor of depressive symptoms. Among the nine studies, five reported a direct prospective association [32, 47, 48, 50, 51], one identified an indirect association mediated by body dissatisfaction [45], and three found no significant association [39, 44, 49]. Goodman & Must [47] found a significant three-year prospective association between severe obesity and depressive symptoms. This association did not vary by gender but was moderated by ethnicity, remaining significant only for White-Hispanic adolescents. Pryor *et al*. [51] found that children’s overweight/obesity trajectories were associated with a risk for depression at age 13 via peer victimization and a desire to be thinner, regardless of gender. Additionally, Martin-Storey & Crosnoe [50] found that stable overweight or obesity trajectories from early childhood over 12 years were associated with higher depressive symptoms for girls but not boys. Similarly, Ames & Wintre [32] found that BMI was positively associated with depressive symptoms at ages 16 and 17, but only for girls. However, Hoare *et al*. [48] found that stable overweight or obesity over two years was associated with an increased likelihood of depressive symptomatology, but only in boys. Blundell *et al*. [45] provide additional insight by demonstrating that body dissatisfaction plays a critical role in the relationship between obesity and depressive symptoms. Specifically, their findings indicate that children with a higher BMI at age 7 experienced significantly greater body dissatisfaction at age 11, which, in turn, predicted higher depressive symptoms at age 14. After adjusting for mediators, the direct association between BMI at age 7 and depressive symptoms at age 14 was no longer significant. In contrast, Huang *et al*. [49], Marmorstein *et al*. [39] and Beltrán-Garrayo *et al*. [44] found no direct association between high BMI trajectories and subsequent depression.

Two studies addressed specific anxiety outcomes. Pryor *et al*. [51] found that children on an early- and late-onset overweight/obesity trajectory were at increased risk for anxiety at age 13, mediated by peer victimization and a desire to be thinner. In contrast, Beltrán-Garrayo *et al*. [44] assessed anxiety but reported no significant longitudinal associations between childhood obesity and later anxiety symptoms.

Two studies assessed anxiety and depression jointly using internalizing measures. Ames & Wintre [32], in addition to their analyses of depressive symptoms, reported that higher BMI was associated with higher levels of internalizing symptoms from early to mid-adolescence for both genders. Patalay and Hardman [40] reported that BMI at age 7 predicted internalizing symptoms at age 11 (*p* < 0.05), but BMI at age 11 did not predict internalizing symptoms at age 14.

### Quality of the Studies

Table 4 (Ref. [31, 32, 33, 34, 35, 36, 37, 38, 39, 40, 41, 42, 43, 44, 45, 46, 47, 48, 49, 50, 51, 52]) presents the results of the assessment of study quality based on the Newcastle-Ottawa Scale criteria [31]. All studies demonstrated adequate representativeness and sample selection. Regarding the measurement of psychological variables, all studies used structured psychological interviews (16/21) or validated self-reported questionnaires (21/21) as an inclusion criterion. In terms of weight status measurement, 17 of the 21 studies employed objective assessments, while 4 relied on self-reported weight and height. Regarding the response variable, 12 of the 21 reviewed studies controlled for baseline levels of outcome variables. All studies adjusted statistical analyses for potential confounding variables and had an adequate follow-up duration, as this was a key inclusion criterion. A total of 11 of the 21 studies achieved an appropriate follow-up rate of greater than 75%. Finally, 15 of the 21 studies considered high-quality according to the NOS criteria (NOS ≥7) and six studies were moderate quality (NOS ≥4) (Table 4).

**Table 4.** **Quality appraisal of the selected studies using the Newcastle - Ottawa Quality Assessment Scale (NOS) (Wells *et al*. (2015) [31])**.

Authors and year	C1	C2	C3	C4	C5	C6	C7	C8	Total NOS score
Al-Shoaibi *et al*., 2024 [43]	1	1	1	1	1	1	1	0	7
Ames & Wintre., 2016 [32]	1	1	1	1	1	1	1	0	7
Anderson *et al*., 2011 [33]	1	1	1	1	1	1	1	0	7
Beltrán-Garrayo *et al*., 2023 [44]	1	1	1	1	1	1	1	1	8
Bjornelv *et al*., 2011 [34]	1	1	1	1	1	1	1	1	8
Blundell *et al*., 2024 [45]	1	1	1	1	1	1	1	1	8
Cho *et al*., 2018 [35]	1	1	1	1	1	0	1	1	7
Christiansen *et al*., 2017 [36]	1	1	1	1	1	1	1	0	7
Francis *et al*., 2020 [46]	1	1	1	0	1	0	1	1	6
Goodman *et al*., 2011 [47]	1	1	1	1	1	0	1	0	6
Hoare *et al*., 2016 [48]	1	1	1	1	1	1	1	1	8
Huang *et al*., 2013 [49]	1	1	0	0	1	1	1	0	5
Larsen *et al*., 2014 [38]	1	1	1	0	1	1	1	0	6
Kubzansky *et al*., 2012 [37]	1	1	1	0	1	1	1	1	7
Marmorstein *et al*., 2014 [39]	1	1	1	0	1	1	1	1	7
Martin-Storey & Crosnoe, 2015 [50]	1	1	0	0	1	1	1	1	6
Patalay & Hardman, 2019 [40]	1	1	1	1	1	1	1	0	7
Pryor *et al*., 2016 [51]	1	1	1	1	1	1	1	0	7
Rehkopf *et al*., 2011 [41]	1	1	0	0	1	1	1	0	5
Wade *et al*., 2017 [42]	1	1	0	1	1	1	1	1	7
Yilmaz *et al*., 2019 [52]	1	1	1	0	1	1	1	1	7

Notes: C1, Criterion 1: Representativeness of the exposed cohort: C2, Criterion 2: Selection of the non-exposed cohort; C3, Criterion 3: Ascertainment of exposure; C4, Criterion 4: Demonstration that outcome of interest was not present at the start of the study; C5, Criterion 5: Comparability of cohorts on the basis of the design or analysis; C6, Criterion 6: Assessment of outcome; C7, Criterion 7: Adequacy of follow-up for outcomes to occur; C8, Criterion 8: Adequacy of follow up of cohorts.

## Discussion

The current study reviewed research from the last 12 years on the prospective association between obesity and depressive, anxiety, and eating symptoms during childhood and adolescence. Twenty-one studies were eligible for the synthesis of information, with 15 considered high-quality studies according to the NOS criteria (≥7).

### Psychological Symptoms to Obesity

In the pathway from psychological symptoms to obesity, our review shows that disturbed eating patterns, particularly restrictive eating, predict prospective increases in BMI in childhood and adolescence [34, 36, 41, 42], similar to findings in adult populations [28]. The mixed results regarding emotional eating suggest that this pattern may become more evident in later developmental stages [36, 41], aligning with previous findings that indicate emotional eating emerges after puberty [53] and serves as a risk factor for weight gain in adults [54].

The results concerning depressive symptomatology are more variable. Four studies reported a prospective association between depressive symptoms and obesity, with stronger evidence for girls [33, 37, 38, 39], while two studies did not [35, 36]. According to Mühlig *et al*. [25], this discrepancy may be due to the association being age- and gender-dependent. In our review, Christiansen* et al*. [36] found no association between depressive symptoms and weight status in children ages 8 to 11, with gender differences emerging only in adolescence. Moreover, previous systematic reviews that did not analyze age effects [11, 23, 24] found no evidence of an association in childhood. It is possible that both the duration of follow-up and the developmental stage at which assessments are conducted play critical roles in detecting these associations. For example, Tanofsky-Kraff *et al*. [55] found that depressive symptoms at ages 6 to 12 did not significantly predict increased BMI four years later. In contrast, Richardson *et al*. [56] found a significant association between late adolescent depressive symptoms and overweight in adulthood, but not between early adolescent depressive symptoms and later overweight. These findings suggest that the impact of depressive symptoms on weight may be more pronounced during specific developmental periods and that follow-up assessments must be timed accordingly.

Studies assessing depressive and anxious symptomatology using a joint measure also revealed mixed results. In line with previous findings, Patalay & Hardman [40] found that internalizing symptoms predicted BMI in adolescence but not in childhood. However, Ames & Wintre [32] found this association only for girls, while Bjornelv *et al*. [34] found no association. Findings from the two studies that employed specific measures for anxiety symptomatology suggest a prospective association with high weight trajectories but not with obesity onset [37, 41]. The lack of longitudinal studies examining anxiety symptoms in adulthood limits direct comparisons [8] but highlights the need for further investigation into the obesity-anxiety link.

Additionally, factors such as the inclusion of other psychological variables (e.g., eating problems) in statistical models [34, 36], and short follow-up durations [34, 35, 36] may explain the lack of significant associations when assessing the predictive value of anxiety and depression on weight status.

### Obesity to Psychological Symptoms

In the pathway from obesity to psychological symptoms, this review found that high weight trajectories during childhood, including the presence of (or tendency toward) obesity, were associated with eating disorder symptomatology in adolescence [43, 44, 46, 52], aligning with results in adult populations [13, 57]. Obesity was prospectively linked to the development of disordered eating behaviors such as binge eating, restrictive eating, and compensatory behaviors, with gender and pubertal status playing a moderating role [46, 52]. Studies with longer follow-up periods, such as Wade *et al*. [42] and Francis *et al*. [46], suggest that the long-term impact of disordered eating on weight gain may not be immediately apparent and could require extended observation to fully capture these complex relationships.

Regarding the pathway from obesity to depressive symptomatology, findings remain mixed. Five studies identified a prospective association, albeit with inconsistent gender effects. Specifically, two studies found an association regardless of gender [47, 51], two found an association only for girls [32, 50], and one only for boys [48]. Meanwhile, two studies reported no association in this pathway [39, 49]. This inconsistency highlights the need to consider potential moderating factors, such as gender and developmental stage, as well as underlying mechanisms that may explain this complex relationship.

Only one study addressed specific anxiety symptoms and found a prospective association [51]. Interestingly, Ames & Wintre [32] examined the prospective association of BMI with both internalizing symptomatology (including both anxious and depressive symptoms), and specific depressive symptomatology. While weight status predicted internalizing symptomatology in both genders, it was associated with depressive symptoms in girls but not in boys. These results align with previous findings in youth [25] and adults [58], reinforcing the growing but still inconclusive evidence linking depressive symptomatology and obesity. Gender differences in these associations may become more apparent from puberty onwards [51], particularly for depressive symptoms [32]. Moreover, BMI and anxiety and depressive symptoms may become increasingly interrelated as children age [40].

### Potential Mechanisms

The mixed findings regarding the pathway from obesity to psychological symptoms, particularly depressive symptoms, suggest the influence of underlying mechanisms and moderating factors. Beyond direct causality, it is crucial to consider shared underlying mechanisms that may contribute to both conditions. Emerging evidence indicates that genetic predispositions, early-life stress, neuroinflammatory processes, and dysregulation of the hypothalamic-pituitary-adrenal (HPA) axis may simultaneously influence both mental health and weight status [59]. Additionally, environmental factors such as weight stigma, socioeconomic adversity, and family dynamics may serve as common determinants shaping both psychological distress and weight trajectories [28, 60].

At the individual level, weight stigma from an early age can prompt children with overweight or obesity to attempt to meet the thin ideal through unhealthy weight control behaviors (e.g., dieting) [61, 62], which are a risk factor for eating disorders [63]. Furthermore, dieting increases the risk of binge eating and subsequent weight gain over time [64], exacerbating self-blame and shame and reinforcing a harmful cycle of emotional distress and unhealthy eating behaviors [65]. Emotional eating also serves as a coping mechanism to manage difficult emotions and may mediate the relationship between depression, anxiety, and obesity [53, 66, 67, 68]. Within the studies included in this review, Pryor *et al*. [51] found that body dissatisfaction and peer victimization mediated the relationship between overweight/obesity and the development of anxiety and depressive symptoms; while Blundell *et al*. [45] highlighted body dissatisfaction as a key factor linking early BMI trajectories to later depressive symptoms. Consistently, additional studies have shown that low self-esteem can mediate the association between physical fitness and depressive symptoms [69], and that physical activity is associated with lower depression and anxiety and higher self-esteem in children and adolescents [70]. Together, these findings underscore the role of psychological distress as a pathway connecting physical and mental health. In fact, broader sociocultural factors—such as stigma, peer pressure, and cultural norms—further contribute to body dissatisfaction and exacerbate its psychological impact [71, 72].

Gender differences continue to play a critical role in these pathways. Several studies included in this review [32, 39, 46, 48, 50, 52] found that gender influences the strength of the relationship between weight status and psychological variables, especially during adolescence. Gender may moderate these associations through various mechanisms. For instance, females experience greater sociocultural pressure to achieve the thin ideal and exhibit higher levels of body dissatisfaction [73], which can lead to heightened psychological distress (e.g., depressive, anxiety, and eating symptoms) when weight standards are not met. This effect may be more pronounced during adolescence, as pubertal development brings changes in body shape and body fat, particularly for girls [74], while body image becomes central to identity formation [75]. Moreover, females are more likely than makes to use eating as an emotional regulation strategy [38, 76], possibly due to greater experiences of psychological distress, which peak during adolescence [77, 78].

Ethnicity may also moderate the relationship between obesity and psychological symptoms, particularly through body image perceptions. Although most research on body dissatisfaction has been conducted in Western countries, cultural differences in body shape and weight preferences appear when assessing minority ethnic groups [79, 80]. For instance, while African American and Hispanic cultures may exhibit greater acceptance of larger body sizes, the thin ideal prevails among females in the US and most European countries [81]. Consistent with this, the findings in this review suggest a stronger association between obesity and psychological symptomatology in non-Hispanic white groups [33, 47].

Finally, socioeconomic status is a shared risk factor for both obesity [41] and psychological distress [82], which might partly explain the co-development of both conditions [40].

### Methodological Considerations

Methodological variability should be considered when interpreting inconsistencies in associations. Comparisons between studies is hampered by differences in study designs and statistical analyses (e.g., regression analyses, structural equation models, and mediation models such as parallel process latent growth analysis). Additionally, while all studies controlled for potential confounding variables, as illustrated in Tables 2,3, these varied among studies. This variability is highly dependent on the follow-up length and the number of assessments; longer follow-ups with more assessments tend to evaluate trajectories more effectively (e.g., Blundell *et al*. [45]). Some studies included covariables in the initial models but did not report how these variables influenced the assessed associations. Furthermore, not all studies conducted subgroup analyses by gender and ethnicity [32, 48], while others omitted these entirely [40, 46], limiting confidence in conclusions regarding gender and ethnic disparities.

Several measurement considerations should also be noted. Weight status was frequently assessed using growth trajectories, reporting both initial values (i.e., intercept) and average rates of BMI changes (i.e., slope) [46]. However, some studies used baseline or follow-up BMI or BMI categories without reporting changes over time [48]. Most studies relied on objective anthropometric data, though four studies employed self-reported anthropometric measures [32, 35, 49, 52]. Regarding psychological variables, self-reported questionnaires were the primary assessment tool, with only one study [39] using clinical interviews. Moreover, a notable gap in the literature is the limited number of longitudinal studies examining the association between anxiety symptoms and obesity [9]. This may stem from methodological challenges, including the differentiation between anxiety and other internalizing disorders, variability in assessment tools, and a historical research focus on depression as the primary psychological correlate of obesity. Furthermore, anxiety symptoms can manifest heterogeneously, influencing eating behaviors, physical activity levels, and physiological stress responses in ways that vary across developmental stages [8, 10]. These complexities contribute to inconsistencies in findings and underscore the need for future research using standardized measures and longer follow-up periods to clarify the directionality of this association. Another methodological concern is the joint assessment of anxiety and depression [32, 34], which makes it difficult to disentangle their specific contributions to obesity risk. However, this approach may still be useful from a preventive standpoint, as internalizing symptoms often co-occur and share common risk factors. In line with this, some studies contributed to more than one outcome category (e.g., depressive, anxiety symptoms, and internalizing symptoms), since they employed separate validated instruments for each construct. We chose to report these studies in all relevant subsections to preserve fidelity to the original operationalization of outcomes. While this approach introduces some conceptual overlap, it provides a more accurate representation of the available evidence and allows for clearer comparison across studies. Finally, due to the heterogeneity of study designs, outcome measures, and the absence of a quantitative meta-analysis, formal statistical assessment of publication bias (e.g., funnel plots or Egger’s test) was not feasible. Nevertheless, the potential for publication bias in the existing literature is acknowledged as a limitation of this review.

### Strengths and Limitations

A key strength of this review is its focus on prospective studies, which provide more robust evidence than cross-sectional studies by establishing a sequential relationship between exposure and outcome [83]. Additionally, this is the first systematic review to examine the bidirectional link between different psychological symptoms and weight status in children and adolescents. Previous reviews primarily focused on a single diagnostic approach [24, 26], whereas, given the high comorbidity of psychological disorders, it is valuable to assess multiple disorders together. Furthermore, the study employed a rigorous research design, with a comprehensive search strategy and an independent review conducted by two researchers.

Nonetheless, this review has limitations. The search strategy did not include the term “internalizing symptoms”, and upon reviewing reference lists, some studies were found to assess depressive and anxious symptomatology under this term in the title or abstract. Consequently, some studies that met the criteria may not have been included. Finally, there may be other biases, such as the predominance of studies conducted in developed countries, which makes it challenging to appraise the role of ethnicity, as well as the potential bias favoring the publication of studies with positive results. A notable strength of this review is the exclusive inclusion of prospective observational studies, which reduces heterogeneity associated with intervention effects and enhances the comparability of findings across studies.

### Future Directions

Few studies estimate risk transitions between symptomatology and weight status across age stages. A meta-analysis identified only three studies, focusing on middle childhood and adolescence [84], and suggests that bidirectional associations may emerge during these developmental stages. Further research should explore earlier developmental stages to clarify how psychological symptoms and obesity interact to increase vulnerability to depression, anxiety, and eating problems.

Several considerations should guide future research. First, long-term studies experience high dropout rates [36, 49], making it essential to manage missing data using appropriate statistical methods (e.g., multiple imputation) [85]. The choice of assessment instruments is also crucial. While structured interviews enhance clarity and reduce social desirability bias [86], self-reported questionnaires are valid, time-efficient tools that may facilitate participation. Similarly, self-reported anthropometric data can be a cost-effective alternative when assessing weight status as a continuous variable, but caution is necessary when categorizing weight, as accuracy decreases with increasing BMI, potentially underestimating overweight and obesity prevalence [87, 88, 89].

Moreover, tracking weight status among children with severe obesity using Z-BMI may be inaccurate due to limitations in converting very high BMIs to z-scores based on growth charts [90]. Given the lack of international consensus on BMI reporting in childhood, researchers are encouraged to include multiple parameters (e.g., absolute and percent change in BMI, change in percent of the 95th percentile of BMI, change in percent of the median), especially in longitudinal data [90]. Alternative measures such as skinfold thickness or body fat percentage should also be considered [91].

To better understand these complex interactions, future research should prioritize study designs that account for shared genetic, environmental, and psychological risk factors, including emotional regulation, self-esteem, and weight stigma. Greater diversity in study samples is also necessary particularly by including research from South America, Africa, and Asia, as well as considering gender identity beyond the non-binary gender identity. Finally, to properly assess causality in early developmental periods, multiple assessments should be conducted between baseline and endpoint.

## Conclusions

This review provides a developmental perspective on previous findings, suggesting that a prospective association between obesity and psychological symptoms may be evident from childhood to adolescence. In particular, it highlights a mutual relationship between eating disorders and obesity, as well as potential prospective link between high weight trajectories and depressive symptomatology. The association from depression to obesity may be stronger in older children, especially girls, while the relationship from obesity to depression remains inconsistent for boys and girls, requiring further research.

This systematic review demonstrates that evidence-based longitudinal research examining associations between obesity and psychological symptomatology in childhood and adolescence is limited, especially for anxiety symptoms. While conclusions should be drawn with caution, this review raises awareness of the inconsistencies and shortcomings of current studies and encourages further research through prospective and longitudinal designs to account for potential confounders and elucidate causal pathways more rigorously. Meanwhile, prevention efforts should adopt an integrated approach that targets common risk factors, such as weight stigma and low body esteem, to more effectively reduce the burden of both obesity and mental health disorders in youth.

## Availability of Data and Materials

All data generated or analyzed during this study are included in this published article.

## Supplementary Material

Supplementary material associated with this article can be found, in the online version, at https://doi.org/10.62641/aep.v53i6.1897.

## Appendix

See Table 5.

**Table 5.** **Quality assessment criteria for observational studies, based on the Newcastle-Ottawa Scale**.

Selection	
1 – Representativeness of the exposed cohort	
	1 = truly representative of the average children/adolescent in the community
	1 = somewhat representative of the average children/adolescent in the community
	0 = selected group of users (e.g., nurses, volunteers)
	0 = no description of the derivation of the cohort
2 – Selection of the non-exposed cohort	
	1 = drawn from the same community as the exposed cohort
	0 = drawn from a different source
	0 = no description of the derivation of the non-exposed cohort
3 – Ascertainment of exposure	
	1 = Psychological symptoms: structured interview or validated psychological self-report measure
	1 = Weight status: objective measure
	0 = No information, non-validated psychological measures or self-reported weight status
4 – Demonstration that outcome of interest was not present at start of study	
	1 = yes
	0 = no
Comparability	
5 – Comparability of cohorts on the basis of the design or analysis	
	1 = study accounts/controls for relevant variables
	0 = no adjustment for potential confounders
Outcome	
6 – Assessment of outcome	
	1 = Psychological symptoms: structured interview or validated psychological self-report measure
	1 = Weight status: objective measure
	0 = No information, non-validated psychological measures or self-reported weight status
7 – Was follow-up long enough for outcomes to occur?	
	1 = yes, at least 2 years
	0 = no
8 – Adequacy of follow-up of cohorts	
	1 = Subjects lost to follow-up unlikely to introduce bias; small number (less than 25%) lost, or description was provided of those lost.
	0 = Follow-up rate <75% and o description of those lost, or no statement
